# Safety of Janus kinase inhibitors compared to biological DMARDs in patients with rheumatoid arthritis and renal impairment: the ANSWER cohort study

**DOI:** 10.1007/s10238-024-01360-w

**Published:** 2024-05-10

**Authors:** Yoichi Nakayama, Akira Onishi, Wataru Yamamoto, Ayaka Yoshikawa, Hideyuki Shiba, Naofumi Yoshida, Yonsu Son, Iku Shirasugi, Toshihisa Maeda, Masao Katsushima, Motomu Hashimoto, Yuki Etani, Tetsu Itami, Yuji Nozaki, Hideo Onizawa, Takayuki Fujii, Kosaku Murakami, Koichi Murata, Masao Tanaka, Shuichi Matsuda, Akio Morinobu

**Affiliations:** 1 Department of Rheumatology and Clinical Immunology, Graduate School of Medicine, Kyoto University, Kyoto, Japan; 2 Department of Advanced Medicine for Rheumatic Diseases, Graduate School of Medicine, Kyoto University, Kyoto, Japan; 3 Department of Health Information Management, Kurashiki Sweet Hospital, Okayama, Japan; 4 Department of Internal Medicine (IV), Osaka Medical and Pharmaceutical University, Osaka, Japan; 5 First Department of Internal Medicine, Kansai Medical University, Osaka, Japan; 6 Department of Rheumatology and Clinical Immunology, Graduate School of Medicine, Kobe University, Kobe, Japan; 7 Rheumatic Disease Center, Orthopaedic Surgery, Matsubara Mayflower Hospital, Kato, Hyogo Japan; 8 Department of Clinical Immunology, Graduate School of Medicine, Osaka Metropolitan University, Osaka, Japan; 9 Department of Musculoskeletal Regenerative Medicine, Graduate School of Medicine, Osaka University, Osaka, Japan; 10 Department of Hematology and Rheumatology, Faculty of Medicine, Kindai University, Osaka, Japan; 11 Center for Cancer Immunotherapy and Immunobiology, Graduate School of Medicine, Kyoto University, Kyoto, Japan

**Keywords:** CKD, Rheumatoid Arthritis, ANSWER cohort, drug retention, Janus Kinase, eGFR

## Abstract

Data on the safety of Janus kinase inhibitors (JAKis) in patients with renal impairment are lacking. This study aimed to investigate the safety of JAKis compared to biological (b) DMARDs in patients with rheumatoid arthritis (RA) and renal impairment. We used a multi-centre observational registry of patients with RA in Japan (the ANSWER cohort). We assessed the drug retention rates of b/targeted synthetic DMARDs with different modes of action (tumour necrosis factor inhibitors (TNFis), immunoglobulins fused with cytotoxic T-lymphocyte antigen (CTLA-4-Ig), interleukin-6 receptor inhibitors (IL-6Ris), and JAKis) in patients with RA stratified by pre-treatment estimated glomerular filtration rate (eGFR) levels. The time to discontinuation of bDMARDs or JAKis was analysed using a multivariate Cox proportional hazards model This study included 3775 patients, who were classified into three groups (the normal group (eGFR ≥ 60 mL/min/1.73 m^2^): 2893 patients; CKDa group (eGFR 45–60 mL/min/1.73 m^2^): 551; and CKDb group (eGFR < 45 mL/min/1.73 m^2^): 331). In the CKDb group, the 12-month drug retention rate due to adverse events (AE) was the lowest in patients treated with JAKi (TNFi: 93.1%; IL-6Ri: 94.1%; CTLA-4-Ig: 92.3%; JAKi: 75.1%). In the normal and CKDa groups, drug retention rates due to AE were similar among patients treated with bDMARDs and JAKi. In contrast, drug retention rates due to inefficacy were similar between bDMARDs and JAKis in all groups. In the Cox-proportional model, in the CKDb group, TNFi, IL-6Ri, and CTLA-4-Ig showed lower incidence of drug discontinuation due to AE than JAKis (TNFi: hazard ratio = 0.23 (95% confidence interval 0.09–0.61), IL-6Ri: 0.34 (0.14–0.81), CTLA-4-Ig: 0.36 (0.15–0.89)). JAKis showed the lowest drug retention due to AE in patients with moderate-to-severe and severe renal impairment (eGFR < 45 mL/min/1.73 m^2^). Physicians should pay more attention to renal function when using JAKis than when using bDMARDs.

**Supplementary Information:**

The online version contains supplementary material available at 10.1007/s10238-024-01360-w.

## Background

A decade ago, the first targeted synthetic disease-modifying antirheumatic drug (tsDMARD), a Janus kinase inhibitor (JAKi), was approved for the treatment of rheumatoid arthritis (RA). In contrast, biological DMARDs (bDMARDs) have a longer history of clinical use in RA. The U.S. Food and Drug Administration approved the first tumour necrosis factor inhibitor (TNFi) to treat RA in 1999, the first cytotoxic T-lymphocyte-associated protein 4 immunoglobulin (CTLA-4-Ig) in 2005, and the first interleukin-6 receptor inhibitor (IL-6Ris) in 2010. Currently, four JAKis are used for the treatment of RA: tofacitinib (TOF), baricitinib (BAR), upadacitinib (UPA), and filgotinib (FIL). In certain Asian countries, peficitinib (PEF) is also used in RA clinical practice. Although there have been several randomized controlled trials (RCTs) and post-marketing surveillance, evidence on the safety of JAKis is relatively lacking compared to that on bDMARDs, owing to the shorter time since the approval of JAKis [1–7].

Renal dysfunction is a common comorbidity that is estimated to affect 10–20% of patients with RA [8–10]. The causes of renal impairment include chronic inflammation, ageing, comorbidities, and medications used to treat RA [11]. Renal impairment limits the use of certain DMARDs, including methotrexate, non-steroidal anti-inflammatory drugs, and JAKis, in patients with RA, mainly due to adverse events (AEs). For example, cytopenia, an AE induced by methotrexate (MTX), occurs more frequently in patients with renal impairment. Therefore, careful attention to kidney function is crucial when administering medications for anti-RA drug therapy.

Among the five classes of JAKi, TOF, BAR, UPA, and FIL are mainly or partially excreted by the kidneys [12–15]. BAR undergoes almost complete renal excretion (approximately 75%) and may accumulate within the bodies of patients with renal impairment [13]. TOF also undergoes partial renal excretion (approximately 30%), and the area under the plasma concentration–time curve from time 0 to infinity (AUC_inf_) of this molecule is approximately 1.4-fold higher in patients with moderate renal impairment than in healthy volunteers (Supplementary Table S1) [12, 16, 17]. FIL is mainly excreted in the urine as the metabolite (approximately > 80%) and AUC_24h_ is 1.4–1.7-fold higher in patients with moderate renal impairment than healthy individuals [14]. Thus, TOF, BAR, and FIL require dose reduction in patients with impaired kidney function, whereas BAR and FIL are contraindicated in patients with severe kidney dysfunction. Although the renal elimination rate of UPA is less than 20%, it has a 1.3-fold higher AUC_inf_ in patients with moderate renal impairment [15, 18]. Therefore, although dose reduction is not required, it is recommended that UPA is cautiously used in patients with severe renal impairment. PEF is predominantly excreted in the faeces, and its mean urinary excretion rate is approximately 10%. Considering PEF, the AUC_inf_ value is lower in patients with renal impairment than that in healthy volunteers [19].

In randomized controlled trials, patients with moderate renal impairment were excluded from the study population (estimated glomerular filtration rate (eGFR) < 60 mL/min for TOF and eGFR or estimated creatinine clearance < 40 mL/min for BAR and FIL) [1, 2, 20]. Although some post-marketing or cohort studies have reported the safety data of JAKi as real-world evidence, there has been no safety evidence focusing on patients with RA and kidney dysfunction [5, 21, 22]. This retrospective multi-centre study aimed to investigate the safety of JAKis compared to bDMARDs in patients with renal impairment in a real-world setting.

## Methods

### Patients

The Kansai Consortium for Well-Being of Rheumatic Disease Patients (ANSWER) cohort is a multi-centre registry of patients with RA in the Kansai District of Japan. Patients' data from nine institutes (Kyoto University, Osaka University, Osaka Medical College, Kansai Medical University, Kobe University, Nara Medical University, Osaka Metropolitan University, Kindai University, and Osaka Red Cross Hospital) were included [23–25]. Patient selection was based on the decision of the attending physician, and data were collected prospectively. The data for this cohort are available from 2009 onwards; however, data between 2013 and 2023 were used for the current analysis because TOF was approved in 2013.

The inclusion criteria were as follows: patients diagnosed with RA according to the 2010 American College of Rheumatology/European League Against Rheumatism classification criteria [26] and initiated treatment with a bDMARD or JAKi. Patients in whom PEF was initiated were excluded, given that PEF is mainly metabolized in the liver and AUC_inf_ in patients with renal impairment is even lower than that of healthy individuals (Supplementary Table S1) [19]. In the main analyses, given that the dosages of TOF, BAR, and FIL need to be adjusted based on renal function, we excluded patients with renal impairment in whom no dose reduction was performed for these drugs in accordance with the manufacturer’s recommendation to elucidate the effect of renal impairment on JAKi in the standard care of RA. In the sub-analysis, we compared patients treated with TOF, BAR, and FIL with dose reduction and those without dose reduction according to renal function to determine whether dose reduction in patients with renal impairment is reasonable in real-world clinical practice.

The following data were collected: baseline demographic variables, such as age, sex, disease duration, titres of rheumatoid factor and anti-cyclic citrullinated peptide antibody, concomitant dosages and ratios of methotrexate and prednisolone, disease activity scores, the number of prior use of b/ts-DMARDs, and serum creatinine (SCr).

### Patient stratification according to baseline eGFR

Patients were stratified according to their baseline eGFR. In the primary analysis, eGFR was calculated using an equation officially approved by the Japanese Society of Nephrology (JSN) based on SCr [27]. For sensitivity analyses, we calculated eGFR using the Japanese coefficient-modified modification of diet in renal disease (MDRD) study equation and the Japanese coefficient–modified Chronic Kidney Disease Epidemiology Collaboration (CKD-EPI) study equation [27].

JSN equation: 194 × (SCr)^−1.094^ × (Age)^−0.287^ × 0.739 (if female).

MDRD equation: 175 × (SCr)^−1.154^ × (Age)^−0.203^ × 0.742 (if female), Japanese Coefficient 0.808.

CKD-EPI equation: 141 × min (SCr/*κ*, 1)^α^ × max (SCr/*κ*, 1)^−1.209^ × 0.993^age^ × 1.018 (if female) (where *κ* is 0.9 for males and 0.7 for females, *α* is − 0.411 for males and − 0.329 for females, min is the minimum of SCr/κ or 1, and max is the maximum of SCr/*κ* or 1), Japanese coefficient 0.813,

Referring to the Kidney Disease Improving Global Outcomes (KDIGO) 2012 CKD stage classification criteria, patients were stratified into the following three groups: ‘normal’ (GFR category G1 and G2; eGFR ≥ 60 mL/min/1.73 m^2^), ‘CKDa’ (GFR category G3a; eGFR 45–60 mL/min/1.73 m^2^), and ‘CKDb’ (GFR category G3b, G4, and G5; eGFR < 45 mL/min/1.73 m^2^) [28].

### Treatment groups, outcome measures, and covariates

TNFis included infliximab, etanercept, adalimumab, certolizumab-pegol, golimumab, and biosimilars. The interleukin-6 receptor inhibitors (IL-6Ris) comprised tocilizumab and sarilumab. The cytotoxic T-lymphocyte-associated protein 4 immunoglobulin (CTLA-4-Ig) used was abatacept, whereas JAKis included TOF, BAR, UPA, and FIL.

As dose adjustment is not required for UPA even in patients with renal impairment, we conducted sensitivity analyses after removing UPA from the JAKi treatment group. In these analyses, JAKis group included TOF, BAR, and FIL with appropriate dose reduction in patients with renal impairment.

The primary outcomes of interest were overall 12-month drug retention rates and 12-month cause-specific retention rates, owing to toxic AEs, both adjusted with potential confounders. The 12-month drug retention rate due to inefficacy was also assessed. The follow-up period was set by considering the number of patients at risk, given that a limited number of patients were treated with JAKi for more than 12 months. Treating physicians were asked to adopt the most appropriate reason for discontinuation from the following five categories: remission, inefficacy, AE, other reasons (e.g. patient preference or economic reasons), and loss to follow-up [21]. Regarding the cause-specific retention rate, we focused on the reasons for drug discontinuation due to inefficacy and AEs after the initiation of bDMARD or JAKi.

A priori confounders included age, RA disease duration, use of methotrexate, use of glucocorticoids, and the number of prior bDMARDs and JAKis.

### Statistical analysis

Confounder-adjusted drug retention curves were generated based on the stratified groups (normal, CKDa, and CKDb). The time to discontinuation of bDMARDs or JAKis was analysed using a multivariate Cox proportional hazards model as a reference for JAKis to determine their safety compared with each bDMARD. A priori confounders included age, RA disease duration, use of methotrexate, use of glucocorticoids, and the number of prior bDMARDs and JAKis. All analyses were two-tailed, and statistical significance was set at *p* < 0.05. All analyses were performed using R Statistical Software (v4.3.1; R Foundation for Statistical Computing, Vienna, Austria).

## Results

### Patient characteristics stratified by kidney function groups

**Fig. 1:**
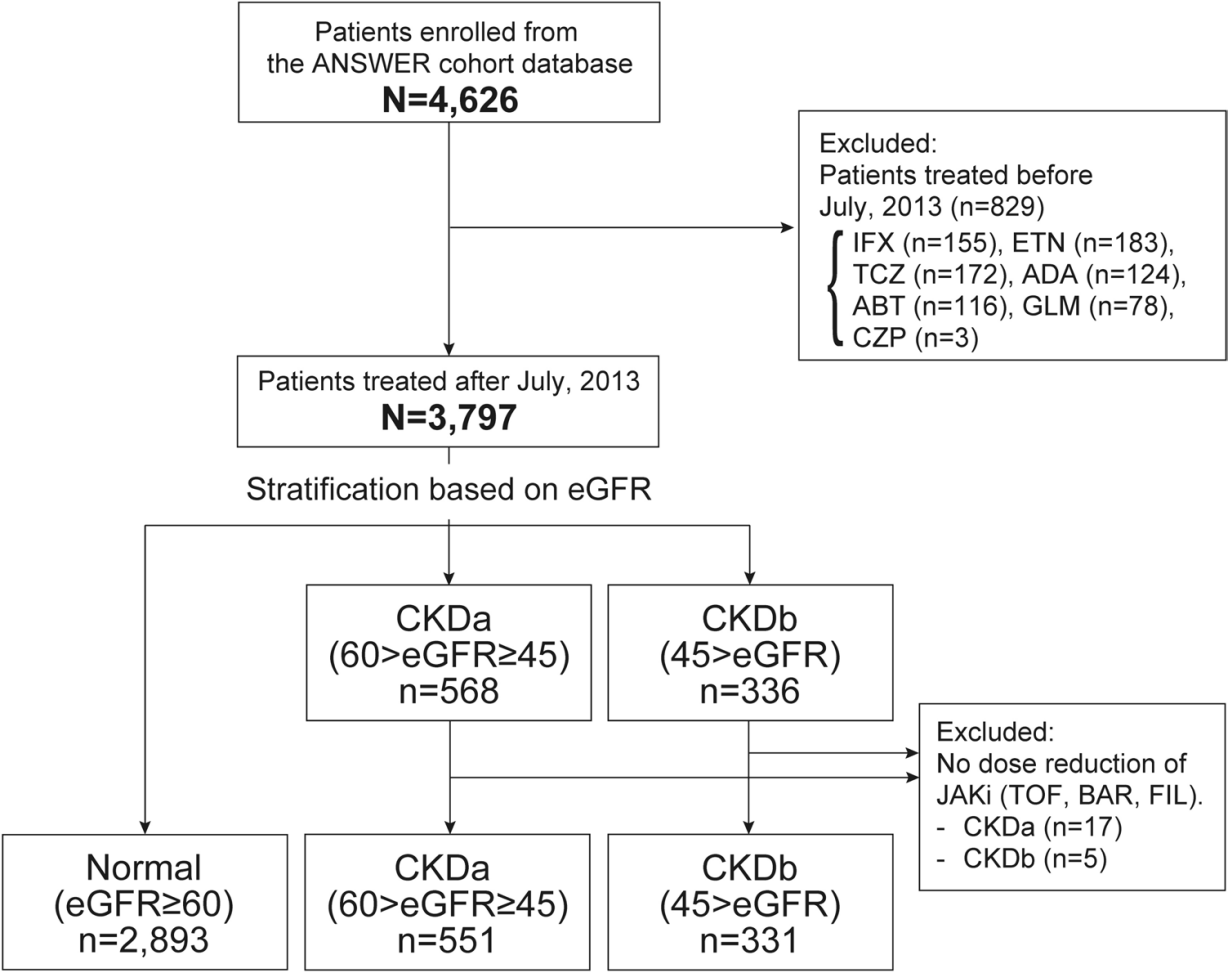
Flow diagram of patients included in this study

This study included 3,775 patients with RA receiving treatment with bDMARDs or JAKis (normal group, 2,893 patients; CKDa group, 551; CKDb group, 331) (Fig. 1). The baseline patient characteristics of the three bDMARDs and JAKis in each kidney function group are shown in Table 1. The proportion of the different JAKis in each group were as follow: in the normal group, TOF 132 (28%), BAR 197 (43%), UPA 76 (17%), FIL 52 (11%); in the CKDa group, TOF 28 (29%), BAR 27 (28%), UPA 29 (30%), FIL 12 (13%); in the CKDb group, TOF 11 (20%), BAR 10 (19%), UPA 23 (43%), FIL 10 (19%). In all groups, JAKis were used less frequently in biologic-naïve patients. Baseline eGFR levels were higher in patients treated with JAKi in the CKDb group.

**Table 1:** Baseline characteristics of patients treated with b/tsDMARDs stratified by pre-treatment estimated glomerular filtration rate

	Normal (eGFR ≥ 60) (*N* = 2893)					CKDa (45 ≤ eGFR < 60) (*N* = 551)					CKDb (eGFR < 45) (*N* = 331)				
	TNFi (*n* = 1289)	IL-6Ri (*n* = 680)	CTLA4-Ig (*n* = 467)	JAKi (*n* = 457)	p-value	TNFi (*n* = 200)	IL-6Ri (*n* = 126)	CTLA4-Ig (*n* = 129)	JAKi (*n* = 96)	p-value	TNFi (*n* = 89)	IL-6Ri (n = 96)	CTLA4-Ig (*n* = 92)	JAKi (n = 54)	*p*-value
Age (Y)	55.8 ± 15.5	59.3 ± 14.8	64.6 ± 11.9	59.1 ± 13.9	< 0.001	70.2 ± 10.0	71.7 ± 9.2	73.5 ± 8.9	70.9 ± 8.8	0.023	73.8 ± 8.1	71.9 ± 11.1	75.0 ± 7.7	74.4 ± 6.6	0.376
Sex, female	1069 (82.9%)	566 (83.2%)	382 (81.8%)	380 (83.2%)	0.926	159 (79.5%)	101 (80.2%)	103 (79.8%)	83 (86.5%)	0.51	71 (79.8%)	73 (76.0%)	69 (75.0%)	43 (79.6%)	0.839
Disease duration (Y)	8.9 ± 9.5	9.8 ± 10.0	10.0 ± 9.7	12.0 ± 9.9	< 0.001	9.7 ± 10.0	11.5 ± 11.5	10.0 ± 9.1	13.4 ± 10.8	0.012	14.5 ± 12.4	14.7 ± 11.6	15.5 ± 13.5	15.8 ± 13.8	0.946
eGFR (mL/min/1.73m^2^)	81.6 (72.3, 95.3)	81.0 (71.2, 95.3)	78.2 (69.0, 90.3)	78.3 (70.6, 89.3)	< 0.001	54.1 (50.9, 57.5)	53.7 (50.3, 56.9)	52.8 (50.0, 56.7)	53.5 (49.5, 56.3)	0.371	37.0 (30.1, 41.6)	35.4 (22.3, 40.9)	36.7 (29.0, 41.2)	40.1 (34.1, 42.1)	0.012
ACPA positive	937 (79.7%)	480 (78.7%)	374 (86.6%)	328 (82.4%)	0.006	132 (74.6%)	82 (75.9%)	97 (86.6%)	68 (81.0%)	0.081	57 (73.1%)	75 (83.3%)	81 (94.2%)	38 (77.6%)	0.003
RF positive	942 (75.1%)	501 (75.8%)	383 (83.4%)	349 (79.0%)	0.002	132 (69.1%)	90 (73.8%)	109 (85.8%)	76 (80.9%)	0.004	64 (72.7%)	76 (80.9%)	79 (88.8%)	42 (77.8%)	0.059
MTX use	892 (69.2%)	348 (51.2%)	201 (43.0%)	264 (57.8%)	< 0.001	111 (55.5%)	38 (30.2%)	48 (37.2%)	33 (34.4%)	< 0.001	22 (24.7%)	22 (22.9%)	20 (21.7%)	10 (18.5%)	0.854
MTX (mg/wk)	10 (8, 12)	8 (6, 12)	8 (6, 12)	8 (6, 12)	0.076	8 (6, 10)	8 (4, 10)	8 (6, 11)	8 (6, 10)	0.66	6 (4, 8)	6 (4, 8)	6 (4, 8)	4 (4, 11)	0.79
PSL use	411 (31.9%)	303 (44.6%)	198 (42.4%)	202 (44.2%)	< 0.001	72 (36.0%)	61 (48.4%)	68 (52.7%)	47 (49.0%)	0.013	47 (52.8%)	46 (47.9%)	39 (42.4%)	29 (53.7%)	0.452
NSAIDs use	710 (55.1%)	394 (57.9%)	213 (45.6%)	272 (59.5%)	< 0.001	95 (47.5%)	62 (49.2%)	60 (46.5%)	50 (52.1%)	0.849	34 (38.2%)	51 (53.1%)	26 (28.3%)	32 (59.3%)	< 0.001
Number of previous b/tsDMARDs					< 0.001					< 0.001					< 0.001
1st	695 (54%)	267 (39%)	295 (63%)	106 (23%)		93 (47%)	49 (39%)	81 (63%)	24 (25%)		42 (47%)	38 (40%)	57 (62%)	8 (15%)	
2nd	345 (27%)	187 (28%)	73 (16%)	106 (23%)		65 (33%)	29 (23%)	29 (22%)	27 (28%)		25 (28%)	29 (30%)	22 (24%)	14 (26%)	
≥ 3rd	249 (19%)	226 (33%)	99 (21%)	245 (54%)		42 (21%)	48 (38%)	19 (15%)	45 (47%)		22 (25%)	29 (30%)	13 (14%)	32 (59%)	
CDAI	14.5 (8.2, 22.3)	16.0 (10.0, 23.0)	16.7 (10.8, 23.5)	15.2 (9.7, 23.4)	0.002	13.2 (7.8, 19.2)	13.1 (9.8, 20.4)	15.0 (10.2, 21.0)	13.8 (10.0, 21.8)	0.587	14.0 (9.0, 22.2)	18.1 (11.4, 26.3)	15.3 (9.5, 19.0)	16.3 (8.5, 25.0)	0.295
HAQ	0.8 (0.3, 1.4)	0.9 (0.4, 1.5)	1.0 (0.4, 1.6)	0.8 (0.3, 1.5)	< 0.001	0.7 (0.3, 1.4)	1.0 (0.4, 1.5)	0.9 (0.3, 1.5)	0.8 (0.4, 1.5)	0.283	1.3 (0.5, 2.0)	1.0 (0.5, 2.0)	1.6 (0.9, 2.1)	1.4 (0.3, 2.0)	0.503
DM	42 (3.3%)	35 (5.1%)	21 (4.5%)	21 (4.6%)	0.197	9 (4.5%)	9 (7.1%)	7 (5.4%)	3 (3.1%)	0.579	9 (10.1%)	18 (18.8%)	11 (12.0%)	4 (7.4%)	0.163
HT	90 (7.0%)	70 (10.3%)	55 (11.8%)	50 (10.9%)	0.003	24 (12.0%)	25 (19.8%)	24 (18.6%)	12 (12.5%)	0.15	22 (24.7%)	36 (37.5%)	28 (30.4%)	12 (22.2%)	0.149

Mean ± SD; n (%); Median (IQR)

*ACPA* anti-citrullinated protein antibody, *b/ts-DMARDs* biological or targeted synthetic disease-modifying antirheumatic drugs, *CDAI* clinical disease activity index, *CTLA4-Ig* immunoglobulin fused with cytotoxic T-lymphocyte antigen, *DM* diabetes mellitus, *HT* hypertension, *IL-6Ri* anti-IL-6 receptor monoclonal antibodies, *JAKi* Janus-kinase inhibitors, *MTX* methotrexate, *NSAIDs* non-steroidal anti-inflammatory drugs, *PSL* prednisolone, *RF* rheumatoid factor, *TNFi* anti-TNF monoclonal antibodies

P value was by intra-group comparison

### Drug retention rates of TNFi, IL-6Ri, CTLA-4-Ig, and JAKi in each eGFR group

**Fig. 2:**
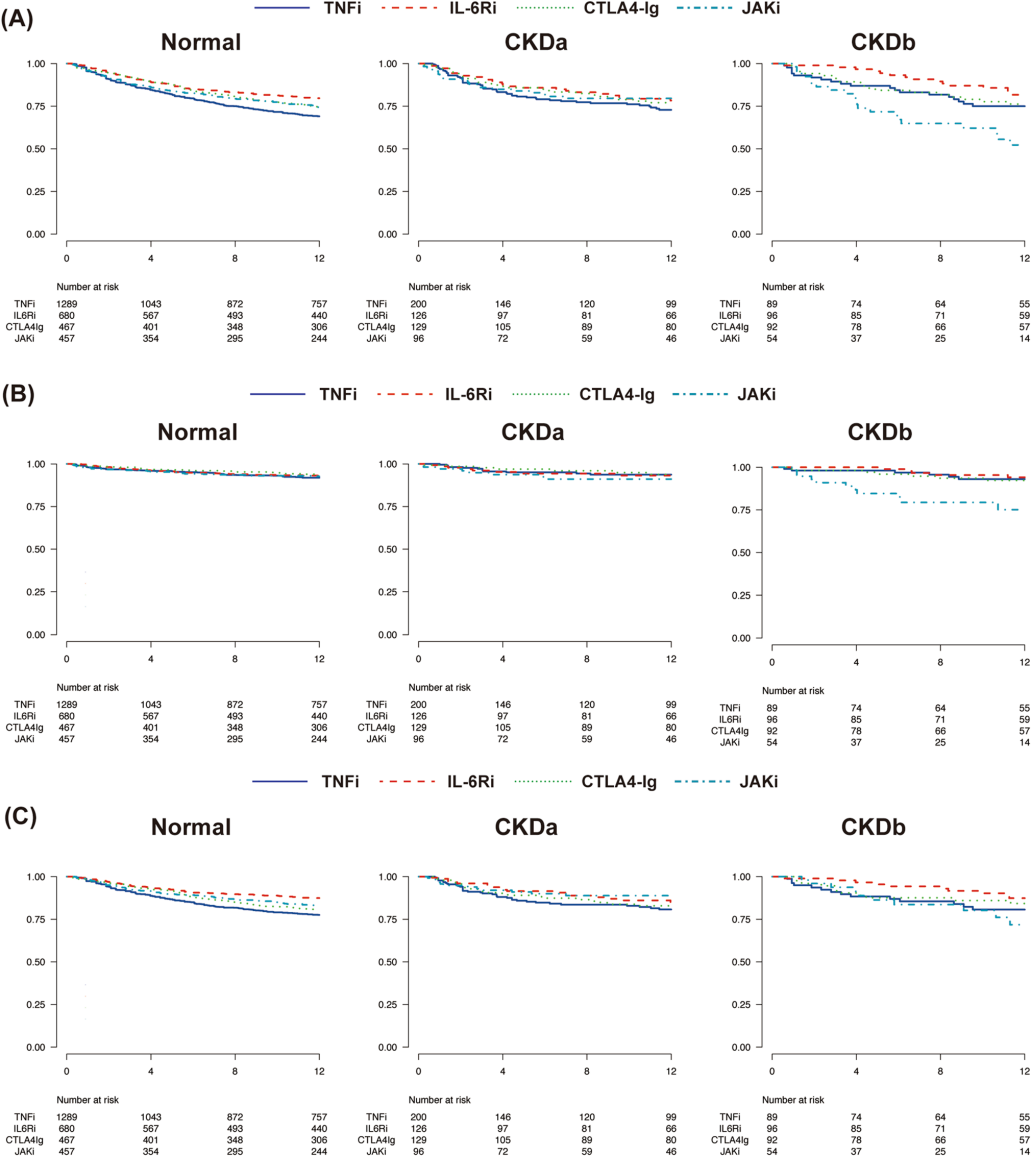
The adjusted 12-month drug retention rate curves. **A** Adjusted overall drug retention rate curves for three categories of bDMARDs and JAK inhibitors (tofacitinib, baricitinib, upadacitinib, and filgotinib) in each eGFR group. The 12-month drug retention rates in the normal group are as follows: TNFi versus IL-6Ri versus CTLA4-Ig versus JAKi (%), 69.1 versus 79.7 versus 74.7 versus 74.4. In the CKDa group, the 12-month drug retention rates are as follows: TNFi versus IL-6Ri versus CTLA4-Ig versus JAKi (%), 72.9 versus 78.3 versus 77.1 versus 79.6. In the CKDb group, the 12-month drug retention rates are as follows: TNFi versus IL-6Ri versus CTLA4-Ig versus JAKi (%), 75.0 versus 81.7 versus 76.2 versus 52.3. **B** Adjusted drug retention rate curves for toxic AEs in each eGFR group. In the normal group, the 12 month drug retention rates are as follows: TNFi versus IL-6Ri versus CTLA4-Ig versus JAKi (%): 91.9 versus 93.1 versus 93.9 versus 92.7. The 12-month drug retention rates in the CKDa group are as follows: TNFi versus IL-6Ri versus CTLA4-Ig versus JAKi (%): 93.7 versus 93.2 versus 93.8 versus 91.1. In the CKDb group, the 12-month drug retention rates are as follows: TNFi versus IL-6Ri versus CTLA4-Ig versus JAKi (%): 93.1 versus 94.1 versus 92.3 versus 75.1. **C** Adjusted drug retention rate curves based on discontinuation due to the inefficacy in each eGFR group. In the normal group, the 12-month drug retention rates are as follows: TNFi versus IL-6Ri versus CTLA4-Ig versus JAKi (%), 77.5 versus 87.4 versus 80.6 versus 82.5. In the CKDa group, the 12-month drug retention rates are as follows: TNFi versus IL-6Ri versus CTLA4-Ig versus JAKi (%), 80.8 versus 85.0 versus 82.9 versus 88.9. In the CKDb group, the 12-month drug retention rates are as follows: TNFi versus IL-6Ri versus CTLA4-Ig versus JAKi (%), 80.7 versus 87.4 versus 84.3 versus 71.8. Abbreviations: *AE* adverse event, *bDMARDs* biological disease-modifying antirheumatic drugs, *CKD-EPI* cytotoxic T-lymphocyte antigen, CTLA-4-Ig; *eGFR* estimated glomerular filtration rate, *DMARDs* disease-modifying antirheumatic drugs, *IL-6Ris* interleukin-6 receptor inhibitors, *JAKis* Janus kinase inhibitors, *tsDMARD* targeted synthetic disease-modifying antirheumatic drug, *TNFIs* tumour necrosis factor inhibitors

We generated confounder-adjusted drug retention curves for bDMARD and JAKi in each kidney function group (Fig. 2). Adjusted overall 12-month drug retention rates are shown in Fig. 2A. In the CKDb group, the 12-month overall drug retention rate was the lowest in patients treated with JAKi, followed by those treated with TNFi, CTLA-4-Ig, and IL-6Ri (TNFi: 75.0%; IL-6Ri: 81.7%; CTLA-4-Ig: 76.2%; JAKi: 52.3%). In the normal group, patients treated with IL-6Ri showed the highest 12-month overall drug retention rate, whereas in the CKDa group, the four categories of DMARDs showed similar drug retention rates. The adjusted 12-month drug retention rates owing to AEs are shown in Fig. 2B. In the CKDb group, the 12-month drug retention rate due to AEs was lowest in patients treated with JAKi (TNFi: 93.1%; IL-6Ri: 94.1%; CTLA-4-Ig: 92.3%; JAKi: 75.1%). In the normal and CKDa groups, drug retention rates due to AEs were similar among patients treated with bDMARDs and JAKis. The adjusted 12-month drug retention rates owing to inefficacy are shown in Fig. 2C. In the CKDa and CKDb groups, patients treated with bDMARDs and JAKis showed similar drug retention rates due to inefficacy.

The results of the multivariate Cox proportional hazards regression analysis are presented in Table 2. In the CKDb group, IL-6Ri and CTLA-4-Ig showed significantly lower overall discontinuation rates than JAKi (IL-6Ri: hazard ratio (HR) = 0.46, 95% confidence interval (CI) 0.26–0.82; CTLA-4-Ig: HR = 0.54, 95% CI 0.30–0.97) and TNFi, IL-6Ri, and CTLA-4-Ig showed lower incidence of drug discontinuation due to AE than JAKi (TNFi: 0.23 (0.09–0.61), IL-6Ri: 0.34 (0.14–0.81), CTLA-4-Ig: 0.36 (0.15–0.89)).

**Table 2:** Hazard ratios (HRs) for the time to discontinuation of bDMARDs and JAK inhibitors (JAKi) (included tofacitinib, baricitinib, upadacitinib, and filgotinib) analysed with a multivariate Cox proportional-hazards model

Events	Normal	eGFR Group	
		CKDa	CKDb
*Overall*			
JAKi	(Ref)	(Ref)	(Ref)
TNFi	1.07 (0.90–1.26)	0.93 (0.62–1.41)	0.58 (0.33–1.02)
IL-6Ri	0.60 (0.50–0.73)***	0.87 (0.56–1.34)	0.46 (0.26–0.82)**
CTLA4-Ig	0.83 (0.68–1.02)	0.85 (0.54–1.32)	0.54 (0.30–0.97)*
*Adverse events*			
JAKi	(Ref)	(Ref)	(Ref)
TNFi	0.90 (0.64–1.27)	0.53 (0.26–1.08)	0.23 (0.09–0.61)***
IL-6Ri	0.71 (0.49–1.03)	0.60 (0.28–1.26)	0.34 (0.14–0.81)*
CTLA4-Ig	0.75 (0.50–1.12)	0.52 (0.24–1.11)	0.36 (0.15–0.89)*
*Inefficacy*			
JAKi	(Ref)	(Ref)	(Ref)
TNFi	1.06 (0.87–1.29)	1.18 (0.69–2.01)	0.91 (0.44–1.91)
IL-6Ri	0.55 (0.43–0.69)***	1.07 (0.61–1.88)	0.57 (0.27–1.23)
CTLA4-Ig	0.91 (0.72–1.15)	1.06 (0.60–1.88)	0.70 (0.31–1.54)

Each eGFR group was categorized based on pre-treatment eGFR calculated with the Japanese-specific formula based on serum creatinine

Normal eGFR group: eGFR ≥ 60 (mL/min/1.73 m^2^), CKDa group: 60 > eGFR ≥ 45 (mL/min/1.73 m^2^), CKDb group: 45 > eGFR (mL/min/1.73 m^2^)

*CTLA4-Ig* the immunoglobulin fused with cytotoxic T-lymphocyte antigen, *IL-6Ri* anti-IL-6 receptor monoclonal antibodies, *TNFi* TNF inhibitor

*p < 0.05, **p < 0.01, ***p < 0.005

Sensitivity analyses after removing UPA from the JAKi treatment group showed similar findings (Supplementary Fig. S1 and Supplementary Table S2). Renal-JAKis (defined as TOF, BAR, and FIL) demonstrated lower drug retention rates owing to AEs than bDMARDs in the CKDb group.

For additional sensitivity analyses, drug retention curves stratified by eGFR using the MDRD and CKD-EPI equations showed similar results (Supplementary Figs. S2, 3). Histograms of eGFR calculated using the three formulas are shown in Supplementary Fig. S4. In the normal and CKDa groups, adjusted drug retention rates owing to AEs were similar between bDMARDs and JAKi, whereas JAKi showed a lower drug retention rate than bDMARDs in the CKDb group.

### The effect of TOF, BAR, and FIL dose reduction

Furthermore, we compared patients with renal impairment who received reduced doses of TOF, BAR, or FIL and those who received these drugs without dose reduction (Not reduced, TOF 10 mg/day, BAR 4 mg/day, FIL 200 mg/day; Reduced TOF 5 mg/day, BAR 2 mg/day, FIL 100 mg/day). The baseline characteristics are described in the Supplementary Table S3. We generated confounder-adjusted drug retention curves owing to AEs (Supplementary Fig. S5). In the CKDa group, there were no differences between patients with and without reduced doses. In the CKDb group, the 12-month drug retention rate due to AEs was lower in patients who received reduced doses than in those without dose reduction (Not reduced: 51.1%; reduced: 80.5%). In the Cox proportional hazards regression analysis, in the CKDb group, patients who received a reduced dose had a lower incidence of drug discontinuation due to AEs than those without dose reduction, although the difference was not statistically significant (HR 0.15, 95% CI 0.016–1.38).

## Discussion

In this study, we compared drug retention rates in patients with RA stratified by baseline eGFR levels. Regarding drug retention rates due to AE, JAKis showed a comparable retention rate with bDMARDs in patients with eGFR 45–60 mL/min/1.73 m^2^ while JAKis had a significantly lower retention rate in patients with eGFR < 45 mL/min/1.73 m^2^. Regarding retention rates due to inefficacy, JAKis showed a similar retention rate to that of bDMARDs in patients with kidney impairment.

From the viewpoint of pharmacokinetics, renal impairment reduces the excretion rate of JAKi with renal excretion properties and sustains higher concentrations of JAKi in the body. Therefore, for certain JAKis, dose reduction is required in patients with renal impairment [29]. In contrast, renal function did not influence the excretion of bDMARDs. Considering that patients with eGFR < 45 mL/min/1.73 m^2^ who received JAKis showed lower drug retention rates due to AE than those with bDMARDs, even though the dose was reduced as indicated, drug concentrations of JAKis may increase and cause further AE. Meanwhile, drug discontinuation rates due to AE were comparable between JAKi and bDMARDs in patients with eGFR 45–60 mL/min/1.73 m^2^; therefore, the effect of renal impairment on drug concentrations of JAKis may be small, and drug concentrations may have not reached harmful levels.

The safety of different doses of JAKi was assessed in Phase II clinical trials [30–35]. In these trials, some patients received doses of JAKi that were higher than the approved standard dose. Patients who received 10 mg TOF twice a day showed a significantly higher rate of infection than those with placebo, while those who received ≤ 5 mg TOF twice a day had a similar rate of infection [31]. Regarding BAR, patients who received 8 mg/day showed more treatment-emergent AE than those who received lower doses [32, 33]. A similar dose-dependent effect was observed in patients administered different doses of UPA. Infection rates, although not serious, were higher in patients who received higher doses of UPA [34, 35]. The results of Phase II clinical trials suggest that higher concentrations of JAKis may increase AE rates.

A safety surveillance study comparing TOF with TNFi was reported in patients with RA who were aged ≥ 50 years and had at least one cardiovascular risk factor [5]. The most frequent AEs were infections. For serious infections, patients who received 10 mg TOF twice a day showed more events than those who received 5 mg TOF twice a day, and TOF had a significantly higher HR than TNFi [36]. In patients aged > 65 years, the HR of all infections and serious infections in patients receiving 10 mg TOF twice daily were higher than those in patients aged 50–65 years old. Since patients with eGFR < 60 mL/min/1.73 m^2^ were excluded from the study, these results were based on patients with normal renal function. In our analysis, the ages of patients in the CKDa and CKDb groups were similar, but the discontinuation rate of JAKis compared to that of bDMARDs increased only in the CKDb group. Although age is associated with more AE, including infections, impaired renal function may also be associated with AE in JAKi compared with bDMARDs.

In Phase II trials of JAKi in patients with RA, drug efficacy was shown to be equivalent between approved and higher doses [30, 32, 35]. In our study, drug efficacy based on drug retention rates was similar between bDMARDs and JAKis in patients with renal impairment. These results imply that the drug concentration of standard-dose JAKis is sufficient in terms of efficacy, and no higher efficacy can be expected even if the JAKi concentration is further increased. Therefore, although the JAKi concentrations were expected to be high in patients with renal impairment, the efficacy of JAKis was unchanged and was comparable to that of bDMARDs in this study.

This study had some limitations. First, the details of hazardous AEs were not evaluated. The attending physicians in our study selected the most suitable grounds for stopping bDMARDs or JAKis from a list that included toxic AE, remission, inefficacy, and others; however, we did not record any particular AE. These may have included drug eruptions, infections, and abnormalities in the laboratory results. Second, as this was a retrospective study, the adjusted models may not have considered additional confounding factors that could affect the drug retention rate. Third, because of the small sample size and lack of significant differences at baseline, we did not include the disease activity index, anti-citrullinated peptide antibody, and rheumatoid factor in the Cox proportional hazards models. Fourth, the number of patients treated with JAKis was lower than those treated with bDMARDs, which could lead to apparently lower drug retention in this group. Fifth, we did not collect data regarding heart failure, renal amyloidosis, and other comorbidities in our cohort. Therefore, there may be insufficient data to identify the study population. Sixth, MTX doses in Japanese patients were lower than those in other countries, and the rate of MTX use may be lower than that in other countries in patients with renal impairment; this could impact the drug-retention rate, and therefore, we adjusted MTX use in our analyses.

## Conclusions

To the best of our knowledge, this is the first study to compare the drug retention rates of bDMARDs and JAKis in patients with RA and pre-existing renal impairment. Compared with bDMARDs, physicians should pay attention to renal function when using JAKis because the AE rate may increase in patients with moderate-to-severe and severe renal impairment (eGFR < 45 mL/min/1.73 m^2^). Further studies with larger sample sizes are required to assess the safety of JAKis in patients with renal impairment.

## Supplementary Information

Below is the link to the electronic supplementary material.

Supplementary file1 (DOCX 16201 KB)

## Data Availability

The datasets used and analysed in the current study are available from the corresponding author upon reasonable request.

## Abbreviations

AE: Adverse event
AUC_inf_: Area under the plasma concentration–time curve from time 0 to infinity
BAR: Baricitinib
bDMARDs: Biological disease-modifying antirheumatic drugs
CKD-EPI: Chronic kidney disease epidemiology collaboration
CTLA-4-Ig: Cytotoxic T-lymphocyte-associated protein 4 immunoglobulin
EGFR: Estimated glomerular filtration rate
FIL: Filgotinib
IL-6Ris: Interleukin-6 receptor inhibitors
JAKis: Janus kinase inhibitors
MDRD: Modification of diet in renal disease
PEF: Peficitinib
RCT: Randomized controlled trial
RA: Rheumatoid arthritis
tsDMARD: Targeted synthetic disease-modifying antirheumatic drugs
TOF: Tofacitinib
TNFIs: Tumour necrosis factor inhibitors
UPA: Upadacitinib
