# Supplementary material for: Safety of Janus kinase inhibitors compared to biological DMARDs in patients with rheumatoid arthritis and renal impairment: the ANSWER cohort study
Source: Clin Exp Med. 2024 May 10;24(1):97. doi: 10.1007/s10238-024-01360-w (PMC11087367; doi:10.1007/s10238-024-01360-w)

**Supplementary Table S1.** The area under the plasma concentration–time curve from time 0 to infinity (AUC_inf_) of each janus kinase inhibitor in individuals with renal impairment compared to healthy volunteers (created by authors).

Mild renal impairment: Cockcroft–Gault creatinine clearance >50 and ≤80 mL/min, Moderate renal impairment: ≥30 and ≤50 mL/min, Severe renal impairment; <30 mL/min.

|  |  | Renal impairment | | |
| --- | --- | --- | --- | --- |
|  | Normal | Mild | Moderate | Severe |
| Tofacitinib (%) | 100 | 137 | 143 | 223 |
| Baricitinib | 1 | 1.41 | 2.22 | 4.05 |
| Upadacitinib (%) | 100 | 118 | 133 | 144 |
| Filgotinib^*^ (%) | 1 | 1.1-1.2 | 1.4-1.7 | 1.5-2.7 |
| Peficitinib | 100 | 87.3 | 83.1 | 108.7 |

^*^ AUC from time 0 to 24 hours.

References
1. Krishnaswami S, Chow V, Boy M, Wang C, Chan G. Pharmacokinetics of tofacitinib, a Janus kinase inhibitor, in patients with impaired renal function and end-stage renal disease. J Clin Pharmacol. 2014;54:46–52. https://doi.org/10.1002/jcph.178

2. Shi JG, Chen X, Lee F, Emm T, Scherle PA, Lo Y, et al. The pharmacokinetics, pharmacodynamics, and safety of baricitinib, an oral JAK 1/2 inhibitor, in healthy volunteers. J Clin Pharmacol. 2014;54:1354–61. https://doi.org/10.1002/jcph.354

3. Namour F, Fagard L, Van der Aa A, Harrison P, Xin Y, Tasset C. Influence of age and renal impairment on the steady state pharmacokinetics of filgotinib, a selective JAK1 inhibitor. Br J Clin Pharmacol. 2018;84:2779–89. https://doi.org/10.1111/bcp.13726

4. Mohamed M-EF, Trueman S, Feng T, Anderson J, Marbury TC, Othman AA. Characterization of the effect of renal impairment on upadacitinib pharmacokinetics. J Clin Pharmacol. 2019;59:856–62. <https://doi.org/10.1002/jcph.1375>

5. Miyatake D, Shibata T, Shibata M, Kaneko Y, Oda K, Nishimura T. Pharmacokinetics and Safety of a Single Oral Dose of Peficitinib (ASP015K) in Japanese Subjects with Normal and Impaired Renal Function. Clin Drug Investig. 2020;40:149-159. https://doi.org/10.1007/s40261-019-00873-7.

**Supplementary Figure S1.** The adjusted 12-month drug retention rate curves.

The category, Renal-JAK inhibitors (Renal-JAKi) include tofacitinib, baricinib, and filgotinib. Each eGFR group is categorized based on pre-treatment eGFR calculated with the Japanese-specific formula based on serum creatinine.

Normal eGFR group: eGFR ≥ 60 (ml/min/1.73m^2^), CKDa group: 60 > eGFR ≥ 45 (ml/min/1.73m^2^), CKDb group: 45 > eGFR (ml/min/1.73m^2^).

Adjusted confounders are baseline age, RA disease duration, the number of previous b/ts-DMARDs exposures, prednisolone use and methotrexate use.

(A). The adjusted overall drug retention rate curve (excluding patient-owed reasons) of three categories of bDMARDs and Renal-JAKi in each eGFR group.

In the normal group, the 12-month drug retention rates are as follows: TNFi vs. IL-6Ri vs. CTLA4-Ig vs. Renal-JAKi (%), 69.0 vs. 79.7 vs. 74.7 vs. 73.2. In the CKDa group, the 12-month drug retention rates are as follows: TNFi vs. IL-6Ri vs. CTLA4-Ig vs. Renal-JAKi (%), 72.5 vs. 78.1 vs. 76.8 vs. 77.1. In the CKDb group, the 12-month drug retention rates are as follows: TNFi vs. IL-6Ri vs. CTLA4-Ig vs. Renal-JAKi (%), 74.3 vs. 81.2 vs. 75.8 vs. 45.9.

(B). The adjusted drug retention rate curve based on discontinuation due to toxic adverse events of three bDMARD categories and Renal-JAKi in each eGFR group.

In the normal group, the 12-month drug retention rates are as follows: TNFi vs. IL-6Ri vs. CTLA4-Ig vs. Renal-JAKi (%), 91.9 vs. 93.2 vs. 93.9 vs. 93.3. In the CKDa group, the 12-month drug retention rates are as follows: TNFi vs. IL-6Ri vs. CTLA4-Ig vs. Renal-JAKi (%), 93.5 vs. 93.1 vs. 93.5 vs. 88.6. In the CKDb group, the 12-month drug retention rates are as follows: TNFi vs. IL-6Ri vs. CTLA4-Ig vs. Renal-JAKi (%), 92.9 vs. 94.0 vs. 92.1 vs. 71.8.

(C). The adjusted drug retention rate curve based on discontinuation due to inefficacy of three bDMARD categories and JAK inhibitors (tofacitinib, baricitinib, upadacitinib, and filgotinib) in each eGFR group.

In the normal group, the 12-month drug retention rates are as follows: TNFi vs. IL-6Ri vs. CTLA4-Ig vs. Renal-JAKi (%), 77.5 vs. 87.4 vs. 80.6 vs. 80.9. In the CKDa group, the 12-month drug retention rates are as follows: TNFi vs. IL-6Ri vs. CTLA4-Ig vs. Renal-JAKi (%), 80.6 vs. 84.8 vs. 82.8 vs. 88.7. In the CKDb group, the 12-month drug retention rates are as follows: TNFi vs. IL-6Ri vs. CTLA4-Ig vs. Renal-JAKi (%), 80.0 vs. 86.7 vs. 83.8 vs. 67.2.

CTLA-4-Ig, the immunoglobulin fused with cytotoxic T-lymphocyte antigen; IL-6Ri, anti-IL-6 receptor monoclonal antibodies; TNFi, TNF inhibitor.


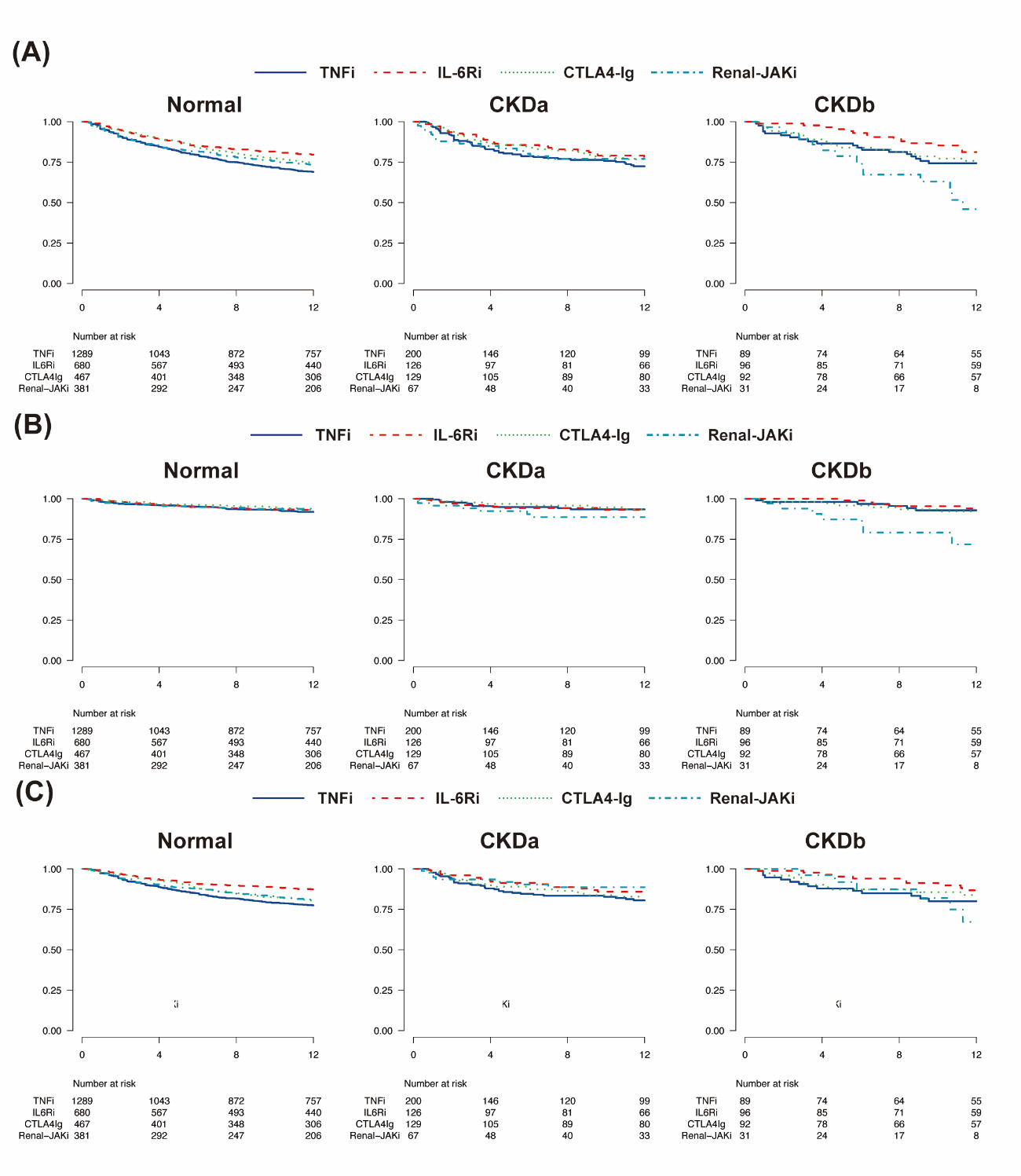


**Supplementary Table S2**. Hazard ratios (HRs) for the time to discontinuation of bDMARDs and Renal-JAK inhibitors (tofacitinib, baricitinib, and filgotinib) analysed with a multivariate Cox proportional-hazards model.

| Events |  |  | eGFR Group |  |
| --- | --- | --- | --- | --- |
| Overall |  | Normal | CKDa | CKDb |
|  | Renal-JAKi | (Ref) | (Ref) | (Ref) |
|  | TNFi | 1.00 (0.84-1.19) | 0.90 (0.57-1.40) | 0.60 (0.31-1.15) |
|  | IL6Ri | 0.56 (0.46-0.68)*** | 0.82 (0.51-1.33) | 0.48 (0.25-0.92)* |
|  | CTLA4Ig | 0.78 (0.64-0.95)* | 0.81 (0.51-1.31) | 0.55 (0.28-1.08) |
| Adverse events |  |  |  |  |
|  | Renal-JAKi | (Ref) | (Ref) | (Ref) |
|  | TNFi | 0.94 (0.65-1.35) | 0.45 (0.22-0.90)* | 0.25 (0.08-0.74)* |
|  | IL6Ri | 0.73 (0.50-1.08) | 0.48 (0.22-1.02) | 0.35 (0.13-0.97)* |
|  | CTLA4Ig | 0.78 (0.51-1.18) | 0.41 (0.19-0.87)* | 0.39 (0.14-1.10) |
| Inefficacy |  |  |  |  |
|  | Renal-JAKi | (Ref) | (Ref) | (Ref) |
|  | TNFi | 0.96 (0.78-1.17) | 1.29 (0.69-2.40) | 0.92 (0.39-2.16) |
|  | IL6Ri | 0.49 (0.39-0.62)*** | 1.17 (0.61-2.24) | 0.58 (0.24-1.40) |
|  | CTLA4Ig | 0.82 (0.65-1.04) | 1.15 (0.60-2.21) | 0.69 (0.28-1.71) |

* p <0.05, ** p <0.01, *** p < 0.005

CTLA-4-Ig, the immunoglobulin fused with cytotoxic T-lymphocyte antigen; IL-6Ri, anti-IL-6 receptor monoclonal antibodies; TNFi, TNF inhibitor.

**Supplementary Figure S2.** The adjusted 12-month drug retention rate curves.

Each eGFR group is categorized based on pre-treatment eGFR calculated with the Japanese coefficient–modified isotope-dilution mass spectrometry-traceable 4-variable Modification of Diet in Renal Disease (MDRD) study equation.

Normal eGFR group: eGFR ≥ 60 (ml/min/1.73m^2^), CKDa group: 60 > eGFR ≥ 45 (ml/min/1.73m^2^), CKDb group: 45 > eGFR (ml/min/1.73m^2^).

Adjusted confounders are baseline age, RA disease duration, the number of previous b/ts-DMARDs exposures, prednisolone use and methotrexate use.

(A). The adjusted overall drug retention rate curve (excluding patient-owed reasons) of three categories of bDMARDs and JAK inhibitors (tofacitinib, baricitinib, upadacitinib, and filgotinib) in each eGFR group.

In the normal group, the 12-month drug retention rates are as follows: TNFi vs. IL-6Ri vs. CTLA4-Ig vs. JAKi (%), 69.2 vs. 79.2 vs. 74.8 vs. 74.1. In the CKDa group, the 12-month drug retention rates are as follows: TNFi vs. IL-6Ri vs. CTLA4-Ig vs. JAKi (%), 73.7 vs. 81.1 vs. 78.7 vs. 82.5. In the CKDb group, the 12-month drug retention rates are as follows: TNFi vs. IL-6Ri vs. CTLA4-Ig vs. JAKi (%), 73.9 vs. 81.6 vs. 73.7 vs. 44.7.

(B). The adjusted drug retention rate curve based on discontinuation due to toxic adverse events of three bDMARD categories and JAK inhibitors (tofacitinib, baricitinib, upadacitinib, and filgotinib) in each eGFR group.

In the normal group, the 12-month drug retention rates are as follows: TNFi vs. IL-6Ri vs. CTLA4-Ig vs. JAKi (%), 92.0 vs. 92.9 vs. 93.8 vs. 92.6. In the CKDa group, the 12-month drug retention rates are as follows: TNFi vs. IL-6Ri vs. CTLA4-Ig vs. JAKi (%), 93.2 vs. 93.8 vs. 95.0 vs. 91.4. In the CKDb group, the 12-month drug retention rates are as follows: TNFi vs. IL-6Ri vs. CTLA4-Ig vs. JAKi (%), 93.9 vs. 95.0 vs. 91.8 vs. 72.4.

(C). The adjusted drug retention rate curve based on discontinuation due to inefficacy of three bDMARD categories and JAK inhibitors (tofacitinib, baricitinib, upadacitinib, and filgotinib) in each eGFR group.

In the normal group, the 12-month drug retention rates are as follows: TNFi vs. IL-6Ri vs. CTLA4-Ig vs. JAKi (%), 77.8 vs. 87.1 vs. 80.7 vs. 82.4. In the CKDa group, the 12-month drug retention rates are as follows: TNFi vs. IL-6Ri vs. CTLA4-Ig vs. JAKi (%), 81.4 vs. 87.0 vs. 84.4 vs. 90.6. In the CKDb group, the 12-month drug retention rates are as follows: TNFi vs. IL-6Ri vs. CTLA4-Ig vs. JAKi (%), 78.5 vs. 86.2 vs. 80.7 vs. 64.2.

CTLA-4-Ig, the immunoglobulin fused with cytotoxic T-lymphocyte antigen; IL-6Ri, anti-IL-6 receptor monoclonal antibodies; JAKi, Janus-kinase inhibitors; TNFi, TNF inhibitor.


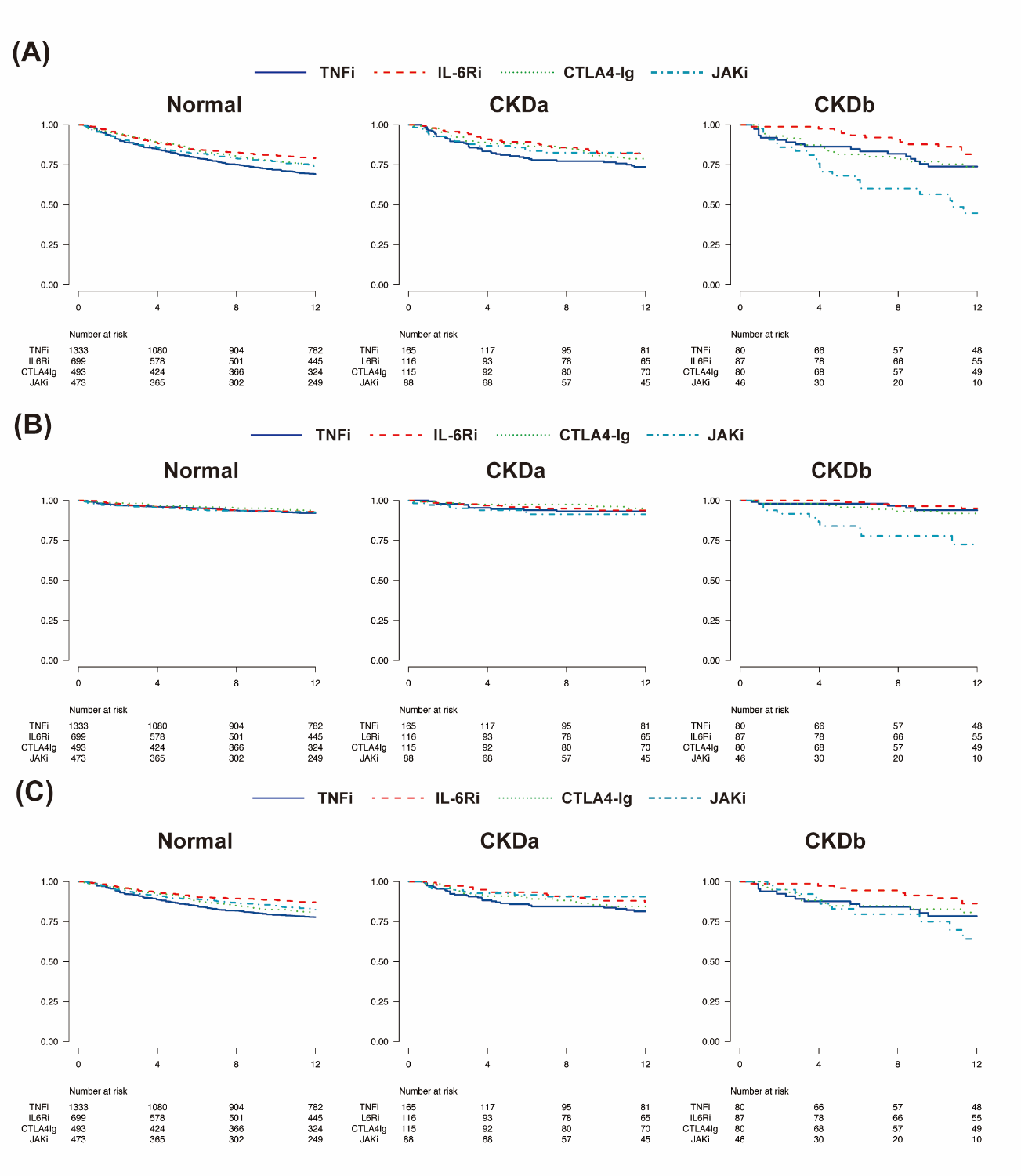


**Supplementary Figure S3.** The adjusted 12-month drug retention rate curves.

Each eGFR group iscategorized based on pre-treatment eGFR calculated with the Japanese coefficient–modified Chronic Kidney Disease Epidemiology Collaboration (CKD-EPI) study equation.

Normal eGFR group: eGFR ≥ 60 (ml/min/1.73m^2^), CKDa group: 60 > eGFR ≥ 45 (ml/min/1.73m^2^), CKDb group: 45 > eGFR (ml/min/1.73m^2^).

Adjusted confounders are baseline age, RA disease duration, the number of previous b/ts-DMARDs exposures, prednisolone use and methotrexate use.

(A). The adjusted overall drug retention rate curve (excluding patient-owed reasons) of three categories of bDMARDs and JAK inhibitors (tofacitinib, baricitinib, upadacitinib, and filgotinib) in each eGFR group.

In the normal group, the 12-month drug retention rates are as follows: TNFi vs. IL-6Ri vs. CTLA4-Ig vs. JAKi (%), 69.5 vs. 79.4 vs. 75.2 vs. 74.7. In the CKDa group, the 12-month drug retention rates are as follows: TNFi vs. IL-6Ri vs. CTLA4-Ig vs. JAKi (%), 73.1 vs. 81.4 vs. 75.4 vs. 72.0. In the CKDb group, the 12-month drug retention rates are as follows: TNFi vs. IL-6Ri vs. CTLA4-Ig vs. JAKi (%), 73.2 vs. 81.3 vs. 75.3 vs. 59.0.

(B). The adjusted drug retention rate curve based on discontinuation due to toxic adverse events of three bDMARD categories and JAK inhibitors (tofacitinib, baricitinib, upadacitinib, and filgotinib) in each eGFR group.

In the normal group, the 12-month drug retention rates are as follows: TNFi vs. IL-6Ri vs. CTLA4-Ig vs. JAKi (%), 92.4 vs. 93.2 vs. 94.2 vs. 92.4. In the CKDa group, the 12-month drug retention rates are as follows: TNFi vs. IL-6Ri vs. CTLA4-Ig vs. JAKi (%), 92.0 vs. 93.9 vs. 92.9 vs. 90.9. In the CKDb group, the 12-month drug retention rates are as follows: TNFi vs. IL-6Ri vs. CTLA4-Ig vs. JAKi (%), 93.3 vs. 94.6 vs. 91.8 vs. 74.3.

(C). The adjusted drug retention rate curve based on discontinuation due to inefficacy of three bDMARD categories and JAK inhibitors (tofacitinib, baricitinib, upadacitinib, and filgotinib) in each eGFR group.

In the normal group, the 12-month drug retention rates are as follows: TNFi vs. IL-6Ri vs. CTLA4-Ig vs. JAKi (%), 77.8 vs. 87.1 vs. 80.8 vs. 83.0. In the CKDa group, the 12-month drug retention rates are as follows: TNFi vs. IL-6Ri vs. CTLA4-Ig vs. JAKi (%), 81.5 vs. 87.3 vs. 82.2 vs. 81.9. In the CKDb group, the 12-month drug retention rates are as follows: TNFi vs. IL-6Ri vs. CTLA4-Ig vs. JAKi (%), 78.1 vs. 86.4 vs. 83.7 vs. 81.7.

CTLA-4-Ig, the immunoglobulin fused with cytotoxic T-lymphocyte antigen; IL-6Ri, anti-IL-6 receptor monoclonal antibodies; JAKi, Janus-kinase inhibitors; TNFi, TNF inhibitor.


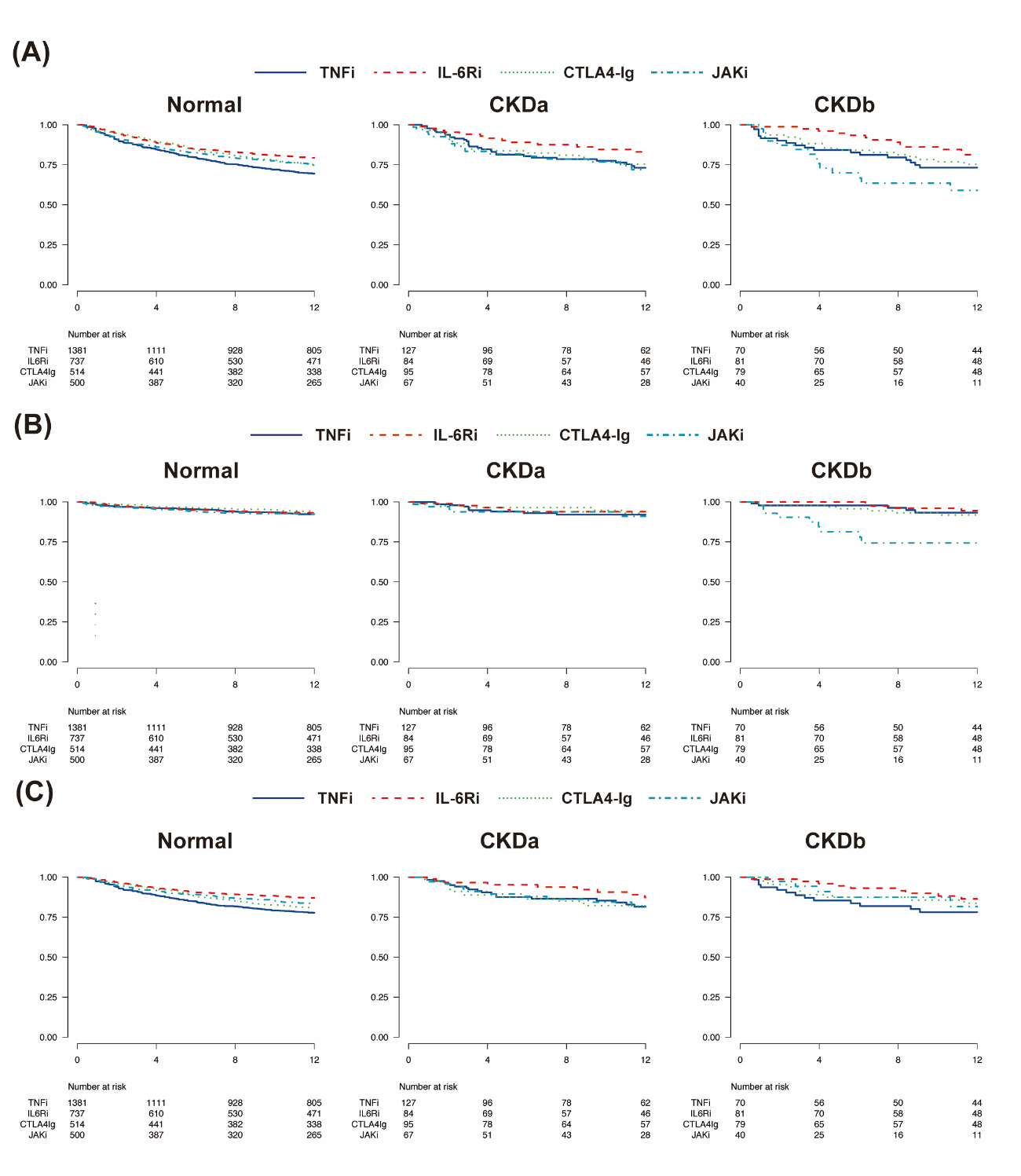


**Supplementary Figure S4.** The histograms of eGFR calculated by the Japanese-specific formula based on serum creatinine (J.CKD), the Japanese coefficient–modified isotope-dilution mass spectrometry–traceable 4-variable Modification of Diet in Renal Disease study equation (MDRD), and the Japanese coefficient–modified Chronic Kidney Disease Epidemiology Collaboration study equation (CKD-EPI).

Mean eGFR are as follows: J.CKD 75.6, MDRD 67.2, and CKD-EPI 75.2 (ml/min/1.73m^2^).

Median eGFR are as follows: J.CKD 74.7, MDRD 66.0, and CKD-EPI 77.4 (ml/min/1.73m^2^)


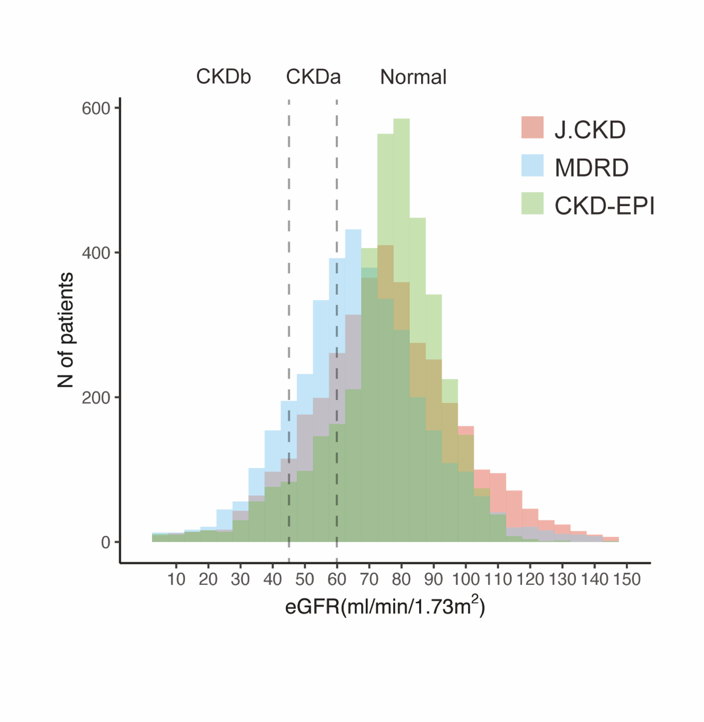


Supplementary Table S3. Baseline characteristics of patients treated with tofacitinib (TOF), baricitinib (BAR), or filgotinib (FIL) with renal impairment (estimated glomerular filtration rate < 60) stratified by dose adjustment (Not reduced, TOF 10mg/day, BAR 4mg/day, FIL 200mg/day; Reduced, TOF 5mg/day, BAR 2mg/day, FIL 100mg/day).

ACPA, anti-citrullinated protein antibody; b/ts-DMARDs, biological or targeted synthetic disease-modifying antirheumatic drugs; CDAI, clinical disease activity index; CTLA4-Ig, immunoglobulin fused with cytotoxic T-lymphocyte antigen; DM, diabetes mellitus; HT, hypertension; IL-6Ri, anti-IL-6 receptor monoclonal antibodies; JAKi, Janus-kinase inhibitors; MTX, methotrexate; NSAIDs, non-steroidal anti-inflammatory drugs; PSL, prednisolone; RF, rheumatoid factor; TNFi, anti-TNF monoclonal antibodies.

P value was by intra-group comparison.

|  | CKDa | | |  | CKDb | | |
| --- | --- | --- | --- | --- | --- | --- | --- |
|  | Not reduced  (N = 17) | Reduced  (N = 67) | P value |  | Not reduced  (N = 5) | Reduced  (N = 31) | P value |
| JAKi |  |  | 0.936 |  |  |  | 0.193 |
| BAR | 8 (47%) | 27 (40%) |  |  | 0 (0%) | 10 (32%) |  |
| FIL | 2 (12%) | 12 (18%) |  |  | 1 (20%) | 10 (32%) |  |
| TOF | 7 (41%) | 28 (42%) |  |  | 4 (80%) | 11 (35%) |  |
| Age (Y) | 65.0 ± 5.9 | 71.7 ± 9.2 | 0.002 |  | 71.6 ± 6.3 | 74.5 ± 6.2 | 0.347 |
| Sex, female | 13 (76.5%) | 58 (86.6%) | 0.288 |  | 4 (80.0%) | 25 (80.6%) | >0.999 |
| Disease duration (Y) | 17.7 ± 13.1 | 12.6 ± 11.4 | 0.078 |  | 12.5 ± 9.1 | 16.1 ± 13.8 | 0.784 |
| eGFR (mL/min/1.73m^2^) | 50.8 (48.9, 53.4) | 54.4 (49.8, 57.0) | 0.014 |  | 34.7 (26.6, 40.2) | 40.5 (35.4, 42.5) | 0.325 |
| ACPA positive | 9 (60.0%) | 49 (81.7%) | 0.091 |  | 4 (80.0%) | 25 (83.3%) | >0.999 |
| RF positive | 10 (62.5%) | 50 (76.9%) | 0.339 |  | 4 (80.0%) | 24 (77.4%) | >0.999 |
| MTX use | 8 (47.1%) | 24 (35.8%) | 0.394 |  | 2 (40.0%) | 7 (22.6%) | 0.581 |
| MTX (mg/wk) | 9 (8, 10) | 8 (6, 11) | 0.55 |  | 8 (8, 8) | 6 (4, 14) | 0.881 |
| PSL use | 9 (52.9%) | 36 (53.7%) | 0.953 |  | 2 (40.0%) | 20 (64.5%) | 0.357 |
| Number of previous b/tsDMARDs |  |  | 0.828 |  |  |  | 0.789 |
| 1 | 3 (18%) | 16 (24%) |  |  | 0 (0%) | 4 (13%) |  |
| 2 | 5 (29%) | 23 (34%) |  |  | 2 (40%) | 7 (23%) |  |
| 3 | 9 (53%) | 28 (42%) |  |  | 3 (60%) | 20 (65%) |  |
| CDAI | 12.1 (6.5, 18.3) | 13.9 (10.0, 24.2) | 0.512 |  | 13.5 (12.7, 18.7) | 18.7 (10.1, 24.5) | 0.763 |
| HAQ | 0.6 (0.3, 2.0) | 0.9 (0.5, 1.8) | 0.554 |  | 0.2 (0.2, 0.2) | 1.6 (0.2, 2.1) | 0.306 |
| DM | 2 (11.8%) | 2 (3.0%) | 0.181 |  | 1 (20.0%) | 2 (6.5%) | 0.37 |
| HT | 5 (29.4%) | 7 (10.4%) | 0.06 |  | 1 (20.0%) | 7 (22.6%) | >0.999 |
| NSAIDs use | 10 (58.8%) | 34 (50.7%) | 0.551 |  | 2 (40.0%) | 19 (61.3%) | 0.63 |

Supplementary Figure S5. The adjusted 12-month drug retention rate curves.

The Not reduced group include patients treated with tofacitinib (TOF), baricitinib (BAR), or filgotinib (FIL) with following doses: TOF 10mg/day, BAR 4mg/day, FIL 200mg/day. The Reduced group include patients treated with following doses: TOF 5mg/day, BAR 2mg/day, FIL 100mg/day.

Each eGFR group is categorized based on pre-treatment eGFR calculated with the Japanese-specific formula based on serum creatinine.CKDa group: 60 > eGFR ≥ 45 (ml/min/1.73m^2^), CKDb group: 45 > eGFR (ml/min/1.73m^2^).

Adjusted confounders are baseline age, RA disease duration, the number of previous b/ts-DMARDs exposures, prednisolone use and methotrexate use.

The adjusted drug retention rate curve based on discontinuation due to toxic adverse events of two categories in each eGFR group.

In the CKDa group, the 12-month drug retention rates are as follows: Not reduced vs. Reduced (%), 93.6 vs. 93.3. In the CKDb group, the 12-month drug retention rates are as follows: Not reduced vs. Reduced (%), 51.1 vs. 80.5.


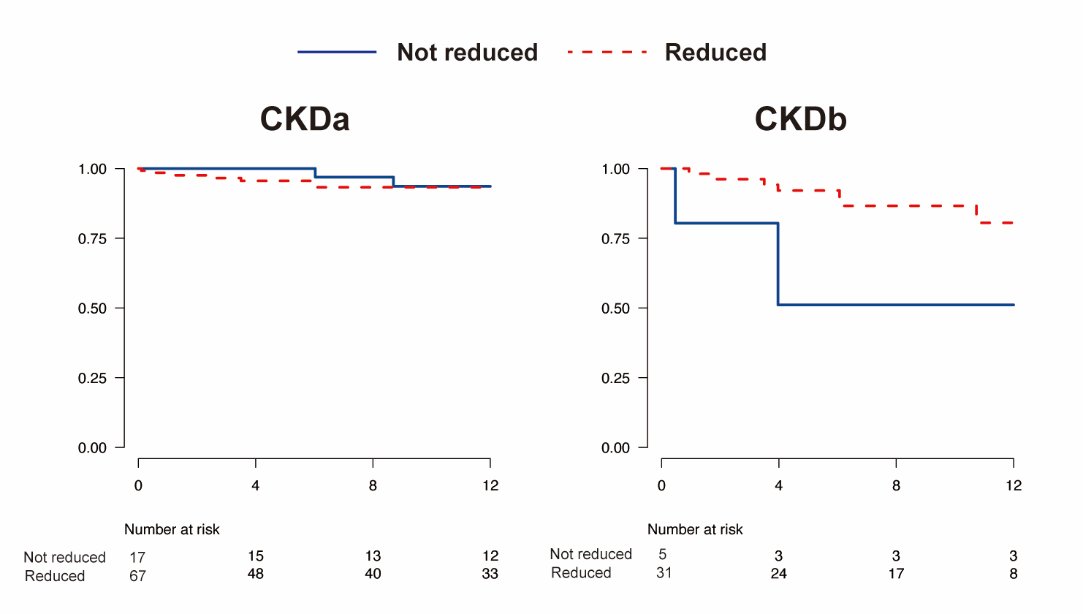

Supplement: Supplementary file 1 — Supplementary file1 (DOCX 16201 KB) [file 10238_2024_1360_MOESM1_ESM.docx]
